# Ultrasound combined with microbubble enhanced renoprotective effects of NLRP3 inflammasome inhibitor MCC950 in CKD model

**DOI:** 10.3389/fphar.2025.1616542

**Published:** 2025-07-31

**Authors:** Wen Wen, Bin Tu, Xiaomei Ren, Yangli Liu, Rufang Jiang, Xiaofeng Wu, Jian Liu

**Affiliations:** ^1^ Department of Ultrasound, Clinical Medical College and The First Affiliated Hospital of Chengdu Medical College, Chengdu, China; ^2^ Department of Ultrasound Medicine, People’s Hospital of Nanbu County, Nanchong, China

**Keywords:** ultrasound, microbubble, CKD, renal fibrosis, NLRP3, MCC950

## Abstract

**Objective:**

This study evaluated the effectiveness of the NLRP3 inflammasome inhibitor MCC950 combined with ultrasound (US) and microbubbles (MBs) on kidney function and fibrosis in a rat model of chronic kidney disease (CKD).

**Methods:**

After establishing the model, SD rats were divided into eight groups (n = 5): Control, CKD, MCC950 (10 mg/kg), MCC950 (5 mg/kg), US + MCC950 (5 mg/kg), US + MBs + MCC950 (5 mg/kg), US and US + MBs. MCC950 was administered at high (10 mg/kg) or low (5 mg/kg) doses, and 200 μL of sulfur hexafluoride microbubbles was delivered via tail vein injection. Ultrasound (mechanical index 0.99) was applied over the kidneys for 10 min every 2 days for six sessions post-injection. Renal function was assessed from urine and blood samples. Kidney tissues were examined using HE and Masson staining, while mRNA and protein levels of NLRP3, caspase-1, ASC, IL-1β, and IL-18 were quantified via RT-qPCR, immunohistochemistry, and ELISA.

**Results:**

Treatment with MCC950 (10 mg/kg), US + MCC950 (5 mg/kg), and US + MBs + MCC950 (5 mg/kg) significantly reduced serum creatinine, blood urea nitrogen, and albumin-to-creatinine ratios, and alleviated kidney damage and fibrosis compared to untreated CKD rats. Notably, US + MBs + MCC950 (5 mg/kg) was as effective as 10 mg/kg MCC950 treatment, with US + MBs further enhancing MCC950’s inhibitory effects on NLRP3 inflammasome activity in renal tissues, manifesting as reductions in the mRNA and protein expression of NLRP3, caspase-1, ASC, IL-1β and IL-18.

**Conclusion:**

The combination of US and MB therapy with MCC950 improves renal function and reduces fibrosis in CKD rats, providing promising evidence for its potential in renal protection and the treatment of inflammatory disorders.

## 1 Introduction

Chronic kidney disease (CKD) is characterized by nephron loss, inflammatory responses, myofibroblast activation, and extracellular matrix deposition. It has emerged as a global public health concern, with its prevalence rising annually in recent years, now affecting approximately 15%–20% of the population worldwide (Matsushita et al., 2010). Patients with end-stage renal disease experience elevated mortality rates and diminished labor productivity, imposing substantial burdens on individuals, families, and society, thereby constituting a severe health challenge (Webster et al., 2017; Yuan et al., 2019). Despite these challenges, effective treatment remains elusive in clinical practice. Renal fibrosis represents a critical pathological condition that profoundly compromises renal function and is noted for its irreversible nature. Inflammation is a fundamental factor in both the initiation and advancement of renal fibrosis (Meng, 2019). Moreover, CKD involves progressive deterioration of renal architecture, glomerular sclerosis, and tubular atrophy, which collectively lead to a decline in renal function over time (Zhang F. et al., 2023; Zheng Y. et al., 2024).

The Nucleotide-binding oligomerization domain (NOD)-like receptor protein 3 (NLRP3) inflammasome, which is composed of NLRP3 protein, Apoptosis-associated Speck-like protein containing a CARD (ASC), and caspase-1, serves as a principal mediator of inflammation associated with CKD, wherein activated NLRP3 inflammasomes facilitate the production and secretion of mature pro-inflammatory cytokines, interleukin-1β (IL-1β) and interleukin-18 (IL-18) (Yuan et al., 2022). Consequently, inhibiting the NLRP3 inflammasome pathway has emerged as a promising therapeutic approach to attenuate renal inflammation and fibrosis. MCC950 is a highly specific small molecule inhibitor of NLRP3 inflammasome activation, derived from diarylsulfonylurea, which has shown efficacy in experimental models and has entered clinical trials (Coll et al., 2019; Tapia-Abellán et al., 2019; Li et al., 2022). While systemic MCC950 administration effectively suppresses inflammation, it is associated with systemic side effects, including hepatic toxicity (Zheng D. et al., 2024). Localized delivery could mitigate these adverse effects but often requires repeated injections and may induce local infections, limiting clinical utility. Therefore, developing targeted delivery systems to enhance MCC950 accumulation specifically in the kidney and allow for lower doses is highly desirable.

Ultrasound (US) technology, historically used for imaging, is rapidly advancing into therapeutic domains due to its ability to deliver focused mechanical energy to tissues, inducing biological effects such as increased cell membrane permeability and cavitation. When combined with microbubbles (MBs), which are gas-filled spheres that can be targeted and ruptured at specific sites, US can significantly enhance cavitation effects, facilitating non-invasive, localized, and controlled drug delivery (Zhang D. et al., 2023). The use of ultrasound-guided microbubbles has shown promise in enhancing the targeted delivery and efficacy of various therapeutics, including anti-inflammatory agents and chemotherapeutics. Research utilizing US and MBs for drug delivery has seen rapid growth in oncology and cardiovascular fields (Ho et al., 2021; Xiang et al., 2021; Han et al., 2022). However, its application in CKD remains limited.

US + MBs approach offers remarkable advantages, such as enhanced local drug concentration, reduced systemic side effects by allowing lower total drug doses, and the potential for precise spatial and temporal control (Roovers et al., 2019; He et al., 2020). Key advantages also include its non-invasiveness, the ability to transiently and reversibly permeabilize biological barriers (e.g., cell membranes, capillary endothelium) at the target site, and the potential for real-time imaging guidance during delivery (He et al., 2022). This localized enhancement leads to improved therapeutic indices, particularly for potent drugs like MCC950 that may have systemic toxicities (e.g., hepatic toxicity with high doses).

However, the application of US + MBs for drug delivery is not without its caveats. Potential disadvantages include the need for careful optimization of ultrasound parameters (frequency, intensity, pulse duration) to achieve optimal therapeutic efficacy while minimizing tissue damage (e.g., thermal effects, mechanical disruption) (Feng et al., 2024). Furthermore, the transient nature of the barrier opening requires precise timing of drug administration. While generally considered safe when parameters are well controlled, potential safety concerns such as microvascular hemorrhage or gas emboli formation (Zhang et al., 2016), although rare, necessitate rigorous pre-clinical validation and careful clinical translation. Thus, a comprehensive understanding of both the immense potential and the inherent challenges is critical for the judicious development and clinical application of US + MBs in disease therapy.

**FIGURE 1:**
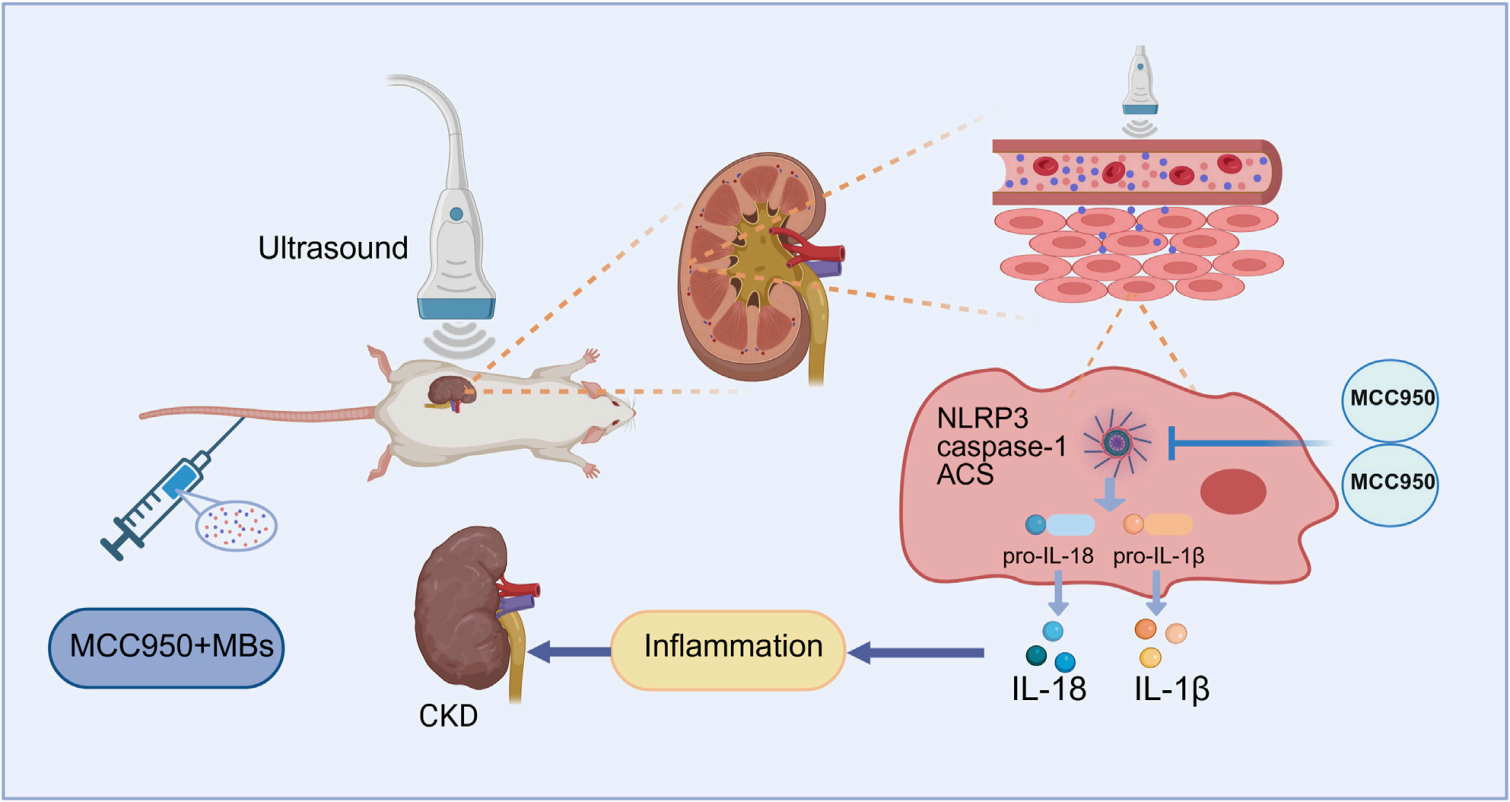
Schematic illustration of the mechanism of MCC950 coupled with ultrasound and microbubble in chronic kidney disease (CKD) therapy. Created with BioRender.com.

Previous studies of our team suggest that ultrasound combined with microbubbles can transiently open biological barriers, increase tissue perfusion, and enable site-specific drug release (Zhang et al., 2025). In this context, we developed an adenine-induced CKD rat model to investigate whether ultrasound combined with microbubbles could enhance the inhibitory effect of low-dose MCC950 on the renal NLRP3 inflammasome. Specifically, we hypothesize that US + MBs may facilitate targeted MCC950 delivery to the kidney, promoting cavitation-mediated permeability and improving therapeutic efficacy, thereby attenuating renal inflammation and fibrosis (Figure 1). This combined approach aims to provide a novel, safe, and effective low-dose therapeutic strategy for CKD.

## 2 Materials and methods

### 2.1 Establishment of CKD rat model

After a 1-week acclimation period, 6–8 weeks male Sprague-Dawley (SD) rats were administered a 2.5% adenine solution (V900471, Sigma, United States) at a dosage of 200 mg/(kg·d) via gavage daily for 28 days, or gavaged with an equal volume of physiological saline to serve as normal controls, with free access to standard chow and water. After 16 days, random urine and peripheral blood samples were collected from the SD rats. Renal function-related urine and serum biochemical parameters were measured using an automated biochemical analyzer to confirm the success of the model before intervention treatment commencing (Li et al., 2024). This study was approved by the Animal Welfare and Ethics Committee of Chengdu Medical College [Chengdu Medical College Ethics (2024) No. 022] and adhered to the 3Rs principle.

### 2.2 Grouping and intervention

**FIGURE 2:**
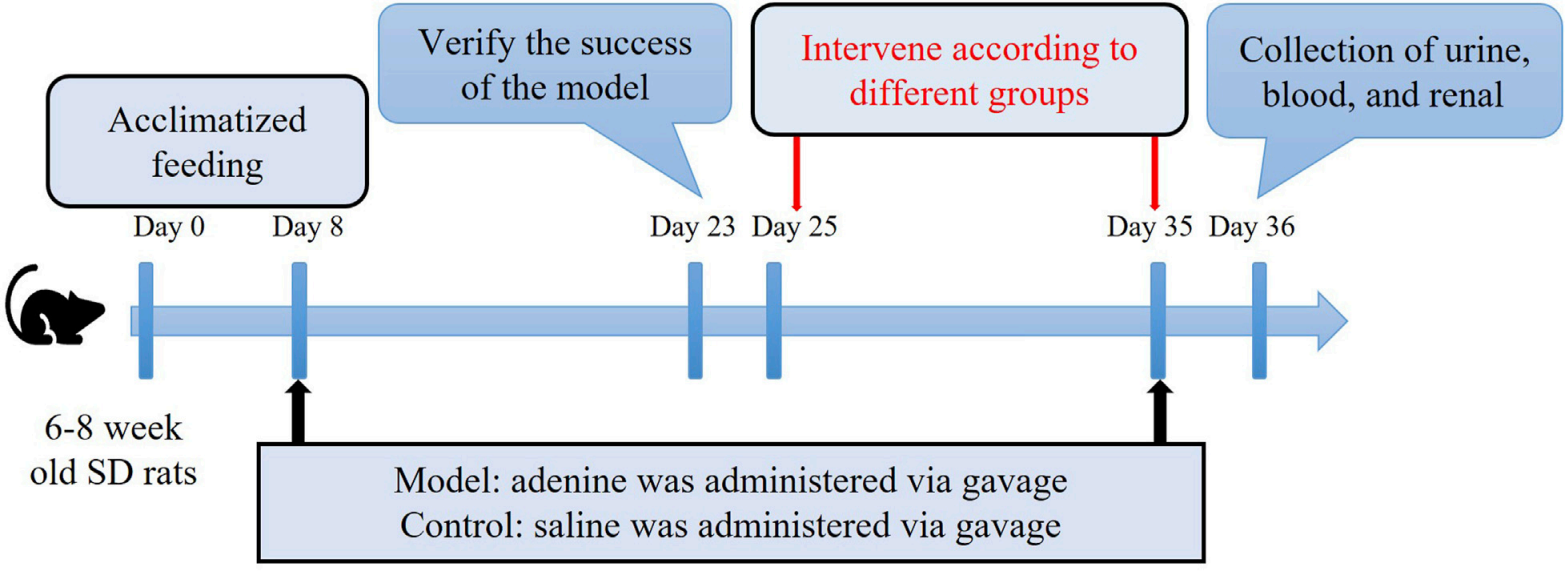
Schematic diagram of the animal research protocol.

Following the successful establishment of the CKD model, the rats were randomly divided into seven groups (n = 4 each) as follows: CKD (no treatment); MCC950 (10) [intravenous injection of 10 mg/kg MCC950 (HY-12815A, MCE, United States)]; MCC950 (5) (intravenous injection of 5 mg/kg MCC950); US + MCC950 (5) (intravenous injection of 5 mg/kg MCC950 combined with ultrasound irradiation towards kidneys); US + MBs + MCC950 (5) (intravenous injection of 5 mg/kg MCC950 and 200 µL of sulfur hexafluoride microbubbles [SonoVue™, Bracco, Italy] combined with ultrasound irradiation); US (ultrasound irradiation); US + MBs (200 µL of sulfur hexafluoride microbubbles combined with ultrasound irradiation). Additionally, five unmodelled SD rats served as the normal control group. The dosage of 10 mg/kg MCC950 used in this study was determined by referring to previous studies (Luo et al., 2019; Zhao et al., 2020). As outlined in Figure 2, the rats in each group received different treatments six times with 48 h intervals.

MCC950 was initially dissolved in dimethyl sulfoxide (D8371, Solarbio, China) to prepare a stock solution, which was then diluted with phosphate-buffered saline (C3580-0500, VC, China) to achieve the required working concentrations (10 mg/kg and 5 mg/kg). Sulfur hexafluoride microbubbles were reconstituted in 5 mL of physiological saline to a concentration of 1–5 × 10^8^ bubbles/mL and were freshly prepared for use. The animal was anesthetized with isoflurane/oxygen gas mixture inhalation (oxygen flow rate 500–800 mL/min, induction concentration 3%–4% and maintenance concentration 1.5%–2.0%). The fur on both sides of the dorsal spine of the rats was shaved to expose the skin overlying the kidneys, and a tail vein access was established.

MCC950 was mixed with sulfur hexafluoride microbubble solution and administered via intermittent injection, immediately following injection, the back skin of the anatomic position of one kidney was exposed to ultrasound irradiation. Cavitation treatment was executed utilizing a commercially available ultrasound cavitation device (G86E, VINNO, China) with an X4-12L linear array probe (frequency 4–12 MHz). The ultrasound parameters employed (Table 1) were optimized in preliminary experiments to achieve effective microbubble destruction and drug delivery to the kidneys while minimizing off-target effects. Taking into account the half-life of sulfur hexafluoride microbubbles (approximately 4–8 min) and the potential for thermal injury to the skin, we first injected half of the solution and immediately applied ultrasound to one kidney for 5 min. Afterward, we injected the remaining and immediately performed ultrasound on the contralateral kidney. The kidneys of the rat measure approximately 1.5–2.3 cm in size and are situated approximately 0.4–0.5 cm beneath the dorsal skin. Therefore, the kidneys, which are the target organs, fall well within the range of ultrasound coverage. Post each cavitation treatment, the skin temperature at the probe contact area on the rats’ backs exhibited a slight increase but normalized before the subsequent cavitation session. No instances of skin damage attributable to temperature increases were observed.

**TABLE 1:** Ultrasound cavitation treatment parameters.

Mechanical index	Frequency	Pulse repetition frequency	Pulse time	Interval time	Total duration	Acoustic power
0.99	3 MHz	200 Hz	2 s	2 s	600 s	70%

### 2.3 Serum renal function and urine albumin to creatinine ratio (ACR) measurement

Following the treatment, all rats underwent a 12-h fasting period with unrestricted access to water. Random urine and peripheral blood samples were subsequently collected. The blood samples were centrifuged at 3,000 rpm for 10 min at 4°C to obtain the upper serum layer. Serum biochemical indicators related to renal function, including serum creatinine (Scr), blood urea nitrogen (BUN), and the urine albumin and creatinine were measured using an automated biochemical analyzer. ACR was calculated using the ratio of urine albumin to urine creatinine.

### 2.4 Renal histological staining

All rats were euthanized with excessive anesthesia and their kidneys were blunt-dissected and placed in pre-cooled physiological saline to remove the renal capsules. Tissues were fixed in 4% paraformaldehyde and then embedded in paraffin. Sections of renal tissue were stained with hematoxylin–eosin (HE) and Masson’s trichrome stains. The histological changes in renal tissue structure and interstitial fibrosis were observed under an optical microscope, with the degree of renal fibrosis assessed using Image J software. The criteria for assessing renal tubular interstitial damage included tubular dilation, loss of brush border, and necrosis/loss of tubular epithelial cells. The scoring system was as follows: 0, no damage; 0.5, <10%; 1, 10%–25%; 2, 25%–50%; 3, 50%–75%; and 4, >75% (Kim et al., 2014).

### 2.5 Immunohistochemical staining

After deparaffinization and hydration, paraffin-embedded renal tissue sections were subjected to antigen retrieval using Trisodium citrate buffer. Following the blockade of endogenous peroxidases, the sections were incubated overnight at 4°C with primary antibodies targeting NLRP3 (1:100, SC06-23, HUABIO, China), IL-18 (1:100, A20473, ABclonal, China), and IL-1β (1:100, BS-0812R, Bioss, China). On the following day, the sections were incubated at 37°C with secondary antibodies (1:100, Servicebio, China) for 20 min, followed by chromogenic detection using DAB, nuclear counterstaining with hematoxylin, dehydration, and slide mounting. Finally, microscopic observation and image capture were performed, and semiquantitative analysis was conducted using ImageJ software.

### 2.6 Real-time quantitative reverse transcription polymerase chain reaction (RT-qPCR)

Total RNA was extracted from kidney tissues according to the reagent kit instructions, reverse transcribed into cDNA, and subsequently amplified. Gene primers were synthesized by Shanghai Bioengineering Co., Ltd., with primer sequences listed in Table 2. Glyceraldehyde-3-phosphate dehydrogenase (GAPDH) was used as the endogenous reference. The relative expression levels of target genes were calculated using the cycle threshold (CT) values from PCR samples, with the relative expression quantified by the 2^−△△CT^ method.

**TABLE 2:** Primer sequences used for Real-time quantitative PCR.

Gene	Forward primer	Reverse primer
NLRP3	5′- GAG​CTG​GAC​CTC​AGT​GAC​AAT​GC -3′	5′- AGA​ACC​AAT​GCG​AGA​TCC​TGA​CAA​C -3′
IL-18	5′- CGA​CCG​AAC​AGC​CAA​CGA​ATC​C -3′	5′- GTC​ACA​GCC​AGT​CCT​CTT​ACT​TCA​C -3′
IL-1β	5′-ATC​CTC​TCC​AGT​CAG​GCT​TCC​TTG​TG -3′	5′- AGC​TCT​TGT​CGA​GAT​GCT​GCT​GTG​A -3′
caspase-1	5′-GAT​GTT​GAC​CTC​AGA​GAA​ATG​AAG​TTG-3′	5′- TGG​GCA​GGC​AGC​AAA​TTC​TTT​C-3′
ASC	5′-CTC​AGA​GCA​GTT​CAT​CGG​CAT​C-3′	5′- GTC​GGT​TCC​AAG​CGT​GTC​ATA​G-3′
GAPDH	5′- ACA​GCA​ACA​GGG​TGG​TGG​AC -3′	5′- TTT​GAG​GGT​GCA​GCG​AAC​TT -3′

GAPDH, glyceraldehyde-3-phosphate dehydrogenase.

### 2.7 Kidney tissue homogenate ELISA

According to the instructions of the kits (ER1094-48T/ER0036-48T, FineTest, China), duplicate wells were set up to measure the expression of IL-18 and IL-1β in rat kidney homogenate. Optical density (OD) was measured at 450 nm using a microplate reader. A standard curve was generated by plotting the OD values of the standards on the X-axis and their concentrations on the Y-axis, with a linear regression equation derived. The OD values of the samples were substituted into the equation to calculate their concentrations.

### 2.8 Statistical analysis

Quantitative data conforming to a normal distribution are presented as mean ± standard deviation. Statistical analysis was performed using GraphPad Prism 9.5.0. Comparisons between two groups were conducted using independent sample t-tests, while comparisons among multiple groups were analyzed using one-way ANOVA. A *P* value of <0.05 was considered statistically significant.

## 3 Results

### 3.1 Enhancement of MCC950 therapeutic effect in CKD rats by ultrasound combined with microbubbles

#### 3.1.1 Comparison of serum scr, BUN and urine ACR in rats

**FIGURE 3:**
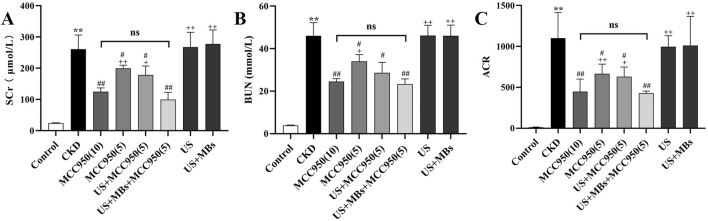
Renal function improvement effects of MCC950 enhanced by ultrasound combined with microbubbles. **(A–C)** Scr, BUN, and ACR in rats after treatment across different groups. *P < 0.05, **P < 0.01 vs. Control group; #*P* < 0.05, ##*P* < 0.01 vs. CKD group; + *P* < 0.05, ++ *P* < 0.01 vs. US + MBs + MCC950 (5) group.

The experimental results (Figure 3) indicated a significant elevation in Scr, BUN levels and urine ACR in the CKD group compared to the control group (*P* < 0.01), signifying severe renal impairment in the CKD rats. In contrast, the serum levels of these indicators were markedly reduced in the MCC950 (10) group, the MCC950 (5) group, the US + MCC950 (5) group and the US + MBs + MCC950 (5) group (*P* < 0.01, respectively) relative to the CKD group, demonstrating a significant improvement in renal function with these treatments. However, there were no significant differences in the aforementioned three indicators between the mechanical control groups (the US group and the US + MBs group) and the CKD group (*P* > 0.05).

Further analysis revealed that there were significant differences in serum Scr, BUN levels, and ACR between the US + MBs + MCC950 (5) group and the MCC950 (5) group (*P* < 0.01). However, no significant differences in serum Scr, BUN levels, and ACR were observed when comparing the US + MBs + MCC950 (5) group with the MCC950 (10) group.

#### 3.1.2 Comparison of HE and masson staining results in rat kidney tissue

**FIGURE 4:**
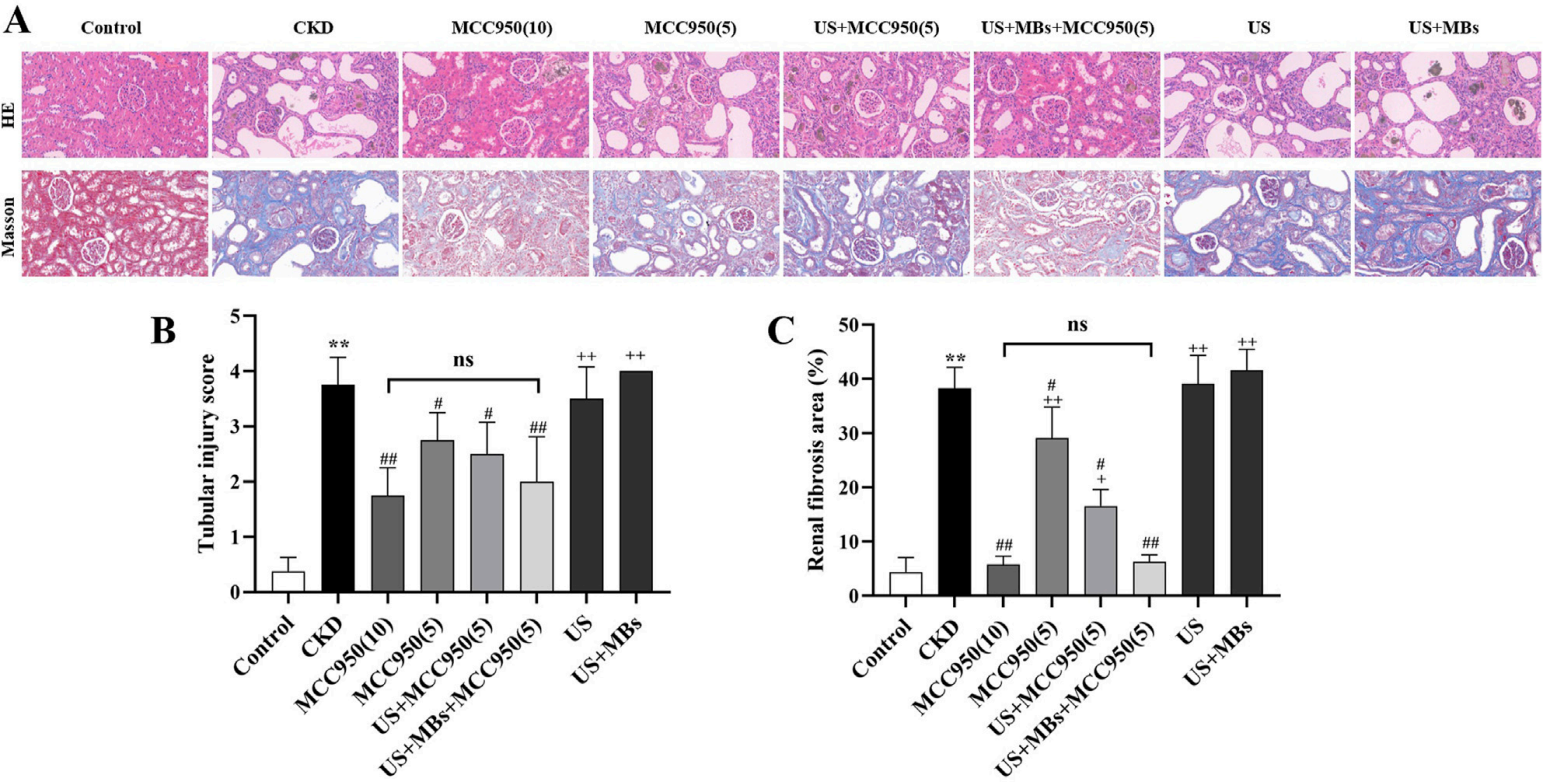
Ultrasound combined with microbubble-enhanced MCC950 ameliorates renal tissue architecture and fibrosis. **(A)** HE and Masson staining of renal tissue paraffin sections (400× magnification); **(B)** Semi-quantitative scoring of tubular injury; **(C)** Quantification of renal interstitial fibrosis area by Image J analysis of Masson-stained sections. *P < 0.05, **P < 0.01 vs. Control group; #*P* < 0.05, ##*P* < 0.01 vs. CKD group; + *P* < 0.05, ++ *P* < 0.01 vs. US + MBs + MCC950(5) group.

HE staining revealed that the kidney tissue structure in the control group was normal, with no apparent abnormalities in the glomeruli, renal tubules, or renal interstitium. Conversely, the CKD group exhibited significant glomeruli reduction and atrophy, pronounced dilation of renal tubules, loss of the brush border, and necrosis/loss of epithelial cells, accompanied by substantial infiltration of inflammatory cells within the renal interstitium (Figure 4A). Quantitative assessment of renal tubular interstitial damage revealed that the CKD group had a significantly elevated score compared to the control group (Figure 4B, *P* < 0.01). However, the groups administered with MCC950 exhibited a marked attenuation in kidney tissue damage (Figure 4A). All four MCC950-treated groups showed a significant reduction in scores (*P* < 0.05 or 0.01), with no statistically significant difference between the group receiving 5 mg/kg MCC950 combined with ultrasound microbubbles and the group receiving 10 mg/kg MCC950 alone (Figure 4B). Overall, treatment with 5 mg/kg MCC950 in combination with ultrasound microbubbles demonstrated superior effects in improving renal tissue structure compared to 5 mg/kg MCC950 alone, and the results were comparable to those observed with 10 mg/kg MCC950 treatment, with both demonstrating inhibition of glomerular atrophy, tubular damage, and inflammatory cell infiltration, but not seen in the groups receiving treatment with US alone (US group) or US combined with MBs (US + MBs group).

Masson staining revealed that the kidney tissue in the control group exhibited red staining with no significant blue staining indicative of fibrosis. In contrast, the CKD group showed extensive blue collagen fiber deposition within the glomeruli and interstitium. Notably, collagen fiber deposition was markedly reduced in all four MCC950-treated groups, with the 10 mg/kg MCC950 alone and the 5 mg/kg MCC950 combined with ultrasound microbubbles groups exhibiting the most pronounced effects (Figure 4A).

Quantitative analysis of collagen fiber area using ImageJ software (Figure 4C) confirmed that the fibrotic area in the CKD group was significantly greater than that in the control group (*P* < 0.01). In comparison to the CKD group, all the groups administered with MCC950 exhibited a significant reduction in fibrotic area (*P* < 0.05 or 0.01), whereas no noticeable improvement was observed in the US group and US + MBs group. Subsequent analyses indicated no significant difference in fibrotic area between the US + MBs + MCC950 (5) group and the MCC950 (10) group. However, the fibrotic area in the US + MBs + MCC950 (5) group was lower than that in the MCC950 (5) group (*P* < 0.01).

### 3.2 Enhanced inhibition of NLRP3 inflammasome by MCC950 combined with ultrasound and microbubbles

#### 3.2.1 NLRP3, ASC, caspase-1, IL-1β, and IL-18 mRNA expressions in rat kidneys

**FIGURE 5:**
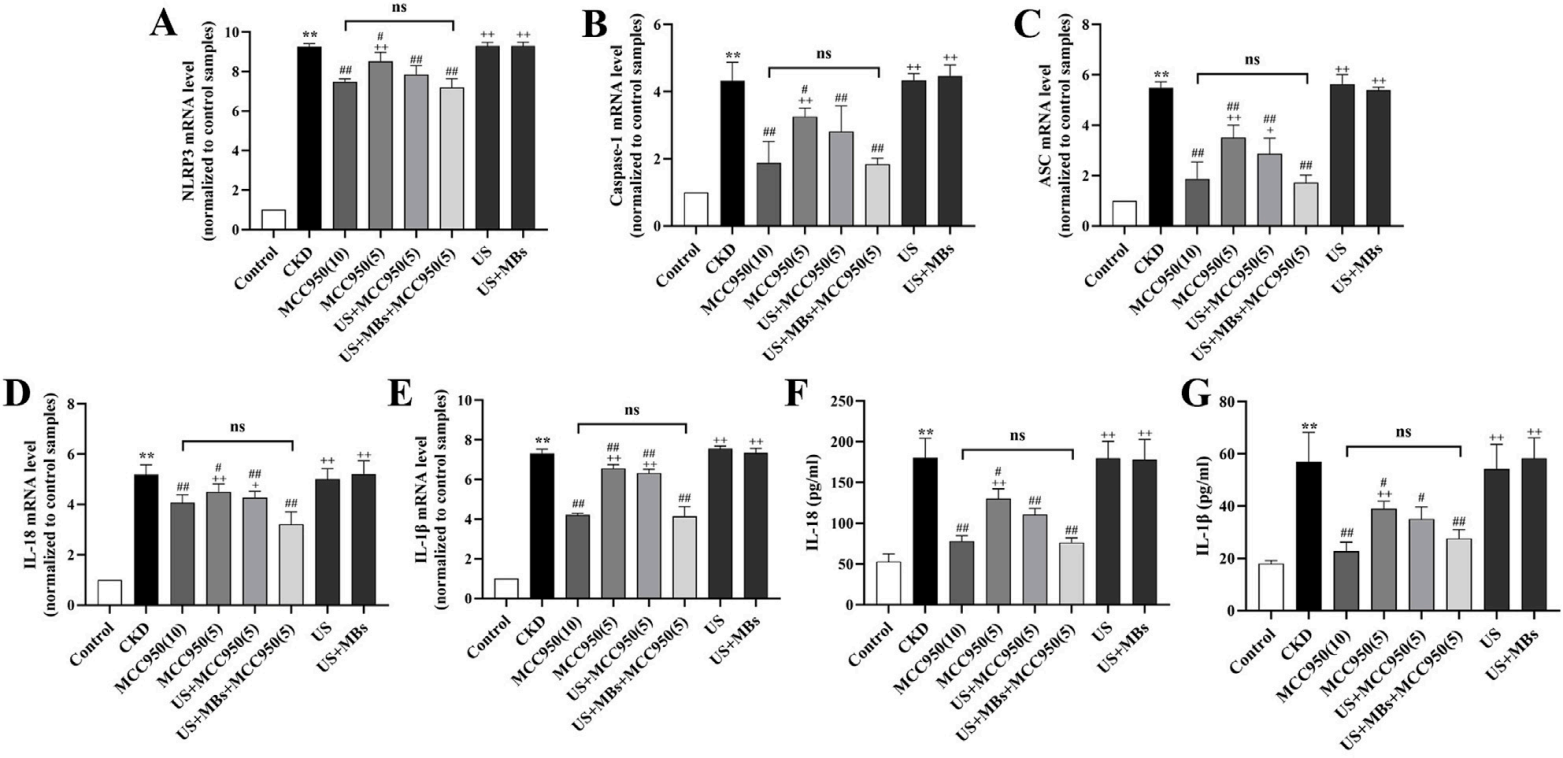
Inhibitory effect of ultrasound combined with microbubble-enhanced MCC950 on NLRP3 inflammasome mRNA expression and IL-18 and IL-1β levels in rat kidney homogenate. **(A–E)** Real-time quantitative PCR (RT-qPCR) analysis of NLRP3, ASC, caspase-1, IL-18, and IL-1β mRNA expression in rat kidney tissues. **(F,G)** ELISA assessment of IL-18 and IL-1β levels in rat kidney homogenate. *P < 0.05, **P < 0.01 vs. Control group; #*P* < 0.05, ##*P* < 0.01 vs. CKD group; + *P* < 0.05, ++ *P* < 0.01 vs. US + MBs + MCC950(5) group.

RT-qPCR analysis (Figures 5A–E) demonstrated that, relative to the control group, the expression of NLRP3, ASC, caspase-1, IL-18, and IL-1β mRNA in the kidneys of CKD rats were markedly elevated (*P* < 0.01). In contrast, treatment with MCC950 resulted in a reduction in the expression of the target genes within renal tissue, with the US + MBs + MCC950 (5) group demonstrating effects analogous to those observed in the MCC950 (10) group. Furthermore, the expression levels of the five mRNAs were lower in the US + MBs + MCC950 (5) group compared to the MCC950 (5) group. However, in the US group or US + MB group, no significant reduction in the aforementioned mRNA levels was observed.

#### 3.2.2 Kidney homogenate IL-18 and IL-1β levels in rats

ELISA analysis of serum IL-18 and IL-1β concentrations (Figures 5F,G) revealed that the levels of IL-18 and IL-1β in the kidney homogenate of CKD rats were significantly elevated compared to the control group (*P* < 0.01). We found that levels of IL-18 and IL-1β were reduced, to different extents, in the four MCC950-treated groups, with no significant difference observed between the US + MBs + MCC950 (5) group and the MCC950 (10) group. Compared to the 5 mg/kg MCC950 group alone, the concentrations of these cytokines were further reduced in the 5 mg/kg MCC950 combined with ultrasound microbubbles group. In contrast, no significant reduction in IL-18 and IL-1β levels was observed in the US and US + MB groups.

#### 3.2.3 NLRP3, IL-18 and IL-1β protein expression in rat kidneys

**FIGURE 6:**
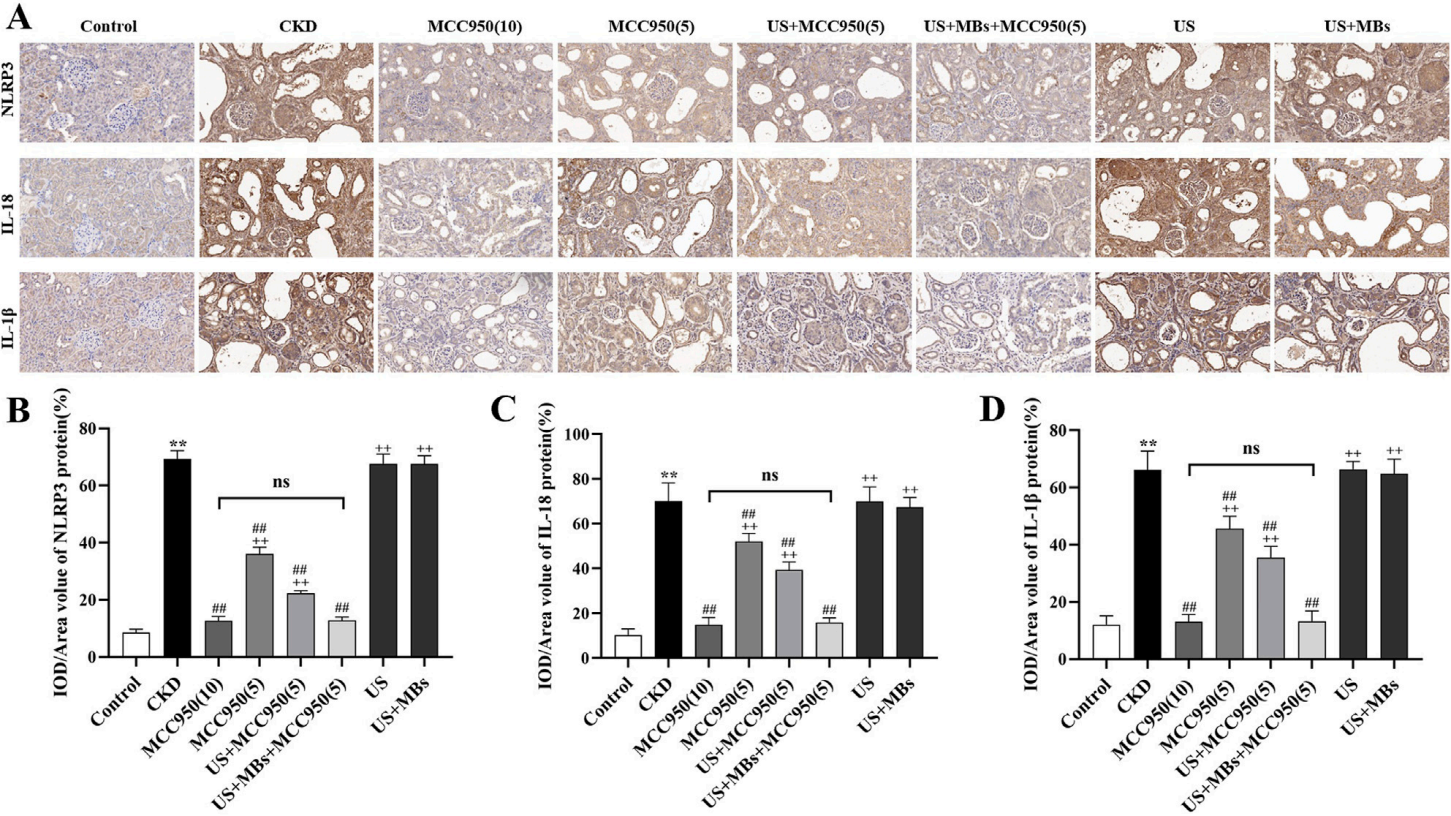
Inhibitory effect of ultrasound combined with microbubble-enhanced MCC950 on NLRP3 inflammasome protein expression. **(A–D)** Immunohistochemical staining of NLRP3, IL-18, and IL-1β protein levels in rat kidney tissues (400× magnification), quantified using ImageJ software. *P < 0.05, **P < 0.01 vs. Control group; #*P* < 0.05, ##*P* < 0.01 vs. CKD group; + *P* < 0.05, ++ *P* < 0.01 vs. US + MBs + MCC950(5) group.

To further corroborate the above findings, immunohistochemical staining was conducted to evaluate the protein levels of NLRP3, IL-18 and IL-1β in rat kidney tissues. The results (Figure 6) demonstrated that the relative expression of NLRP3, IL-18 and IL-1β protein was significantly elevated in the kidneys of CKD rats compared to the control group (*P* < 0.01). In contrast, these proteins levels were significantly reduced in MCC950-treated groups (*P* < 0.01), and the US + MBs + MCC950 (5) group exhibited significantly better results compared to the MCC950 (5) group (*P* < 0.01), while no significant difference was observed between this group and the MCC950 (10) group. No reduction in the levels of these proteins was observed in the groups treated with US or US + MB alone.

## 4 Discussion

In this study, we explored a novel CKD treatment strategy that leverages ultrasound to transition its application from diagnosis to therapy. The results indicated that low-dose MCC950 combined with ultrasound and microbubbles provided superior renal protection compared to low-dose MCC950 alone and was comparable to high-dose MCC950 injections. This was evidenced by reductions in Cr, BUN, and urine ACR, as well as the alleviation of renal structural damage and fibrosis. Further investigation revealed significant reductions in the expression of NLRP3, ASC, caspase-1, IL-18, and IL-1β mRNA, as well as NLRP3, IL-18, and IL-1β proteins in renal tissues, accompanied by corresponding decreases in kidney homogenate IL-18 and IL-1β levels. To our knowledge, this study is the first to demonstrate that ultrasound combined with microbubbles can enhance the efficacy of NLRP3 inflammasome inhibitors, resulting in improved renal function and reduced fibrosis in a CKD rat model.

CKD is a multifactorial condition linked to decreased quality of life, high management costs, and increased mortality (D'Onofrio et al., 2017; Simeoni et al., 2017; Inatomi and Honma, 2023). Inflammation plays a central role in CKD progression, contributing to complications such as anemia, vascular calcification, hypertension, and cardiovascular disease, which impair patient outcomes. Inflammatory cytokines promote renal injury by accumulating in damaged tissues, leading to extracellular matrix deposition and renal fibrosis—the hallmark of CKD progression—culminating in declining renal function (Fan et al., 2014). Current pharmacotherapies like statins exert renoprotective effects by reducing inflammation, oxidative stress, and fibrosis, with emerging agents such as IL-6 inhibitors and SGLT2 inhibitors showing promise in clinical trials (Amann and Benz, 2011; Krane and Wanner, 2011; Razuk et al., 2022; Singhal and Hechanova, 2022; Yuan et al., 2022). Targeting inflammatory pathways offers significant therapeutic potential for slowing CKD progression and improving patient prognosis. Central to this process is the NLRP3 inflammasome, a multiprotein complex expressed in renal cells that detects damage signals like reactive oxygen species (ROS) and ion fluxes, activating caspase-1 to produce pro-inflammatory cytokines IL-1β and IL-18. This cascade further stimulates NF-κB signaling, mitochondrial ROS generation, and lipid accumulation, driving inflammation and fibrosis (Mangan et al., 2018; Li and Chang, 2021). Therefore, modulating inflammasome activation may provide a strategic avenue for CKD intervention. Recent research has focused on the development of novel pharmacological agents targeting the NLRP3 inflammasome signaling pathway. MCC950, first reported in Nature Medicine in 2015 (Coll et al., 2015), is a selective inhibitor of the NLRP3 inflammasome that has exhibited significant anti-inflammatory and protective effects across various disease models. Studies have demonstrated that MCC950 effectively lowers blood pressure in hypertensive murine models and mitigates renal inflammation, fibrosis, and dysfunction (Krishnan et al., 2019). Notably, even when MCC950 treatment is initiated post-onset of proteinuria, mice with apolipoprotein L1-mediated renal injury demonstrate reduced proteinuria and improved renal function (Wu et al., 2021). Furthermore, research by Fu et al. indicates that MCC950 mitigates proteinuria and renal lesions in lupus nephritis mice (Fu et al., 2017). Consistent with previous studies, the present research demonstrates that MCC950, both in combination with ultrasound and microbubbles and as a standalone treatment, effectively inhibits the NLRP3 inflammasome. This is evidenced by the decreased mRNA expression of NLRP3, ASC, caspase-1, IL-18, and IL-1β, as well as reduced protein levels of NLRP3, IL-18, and IL-1β. Consequently, renal inflammation is attenuated, as indicated by lower IL-1β and IL-18 concentrations, ultimately leading to improved renal function in rats with chronic kidney disease, as reflected by a significant reduction in Scr, BUN levels, and ACR.

Although a 10 mg/kg dosage of MCC950 is frequently employed in the literature (Luo et al., 2019; Zhao et al., 2020), higher doses may be linked to adverse effects. Notably, MCC950 underwent clinical phase II trials for rheumatoid arthritis but was discontinued due to elevated serum liver enzyme levels (Mangan et al., 2018; Gao et al., 2023). Therefore, the invstigation of strategies to enhance the efficacy of low-dose MCC950 is of considerable importance. Compared to conventional treatment modalities, the combination of ultrasound and microbubbles presents numerous advantages, including targeting precision, versatility, and safety. Recent experimental studies have increasingly focused on innovative drug delivery systems utilizing ultrasound and microbubbles, with significant progress in cancer therapy (Ho et al., 2021; Xiang et al., 2021; Han et al., 2022). The underlying mechanism of ultrasound combined with microbubbles is predominantly based on their cavitation effects at the disease site. Ultrasound cavitation involves the use of high-intensity ultrasound waves to generate microbubbles within a liquid medium, and the oscillation and subsequent collapse of these bubbles facilitate therapeutic outcomes. One of the primary mechanisms of ultrasound cavitation is its mechanical effects. When subjected to ultrasound, bubbles within the liquid experience cycles of expansion and contraction. At sufficiently high ultrasound intensities, these bubbles abruptly collapse, generating potent mechanical shock waves and shear forces (Mancia et al., 2017; Bouakaz and Michel Escoffre, 2024). These shock waves and shear forces can induce minor damage to the microvascular endothelium, triggering a sterile inflammatory response that results in vasodilation, and consequently enhances blood flow perfusion. Additionally, they can enhance the permeability of cell membranes, facilitating the entry and absorption of drug molecules, thereby improving therapeutic efficacy (Tharkar et al., 2019; He et al., 2022). In this study, ultrasound and microbubble mediated drug delivery enhances the cellular uptake of MCC950 through a combination of physical mechanisms. The cavitation process results in the formation and subsequent disruption of microbubbles within the targeted renal tissue, producing powerful mechanical forces including shock waves and microjets. The transient nature of these forces creates transient pores in the cell membranes, thereby facilitating the direct entry of MCC950 into the cells, a process that bypasses conventional endocytosis-mediated internalization pathways (Geers et al., 2011; Meng et al., 2019).

Beyond enhanced drug penetration, the mechanical stimulation from the cavitation process is capable of modulating intracellular signaling pathways, which may contribute to the observed therapeutic effects. For example, cavitation of oxygen-carrying microbubbles (O_2_-MBs) induced by ultrasound can enhance mechanical forces on endothelial cells. These forces can trigger endothelial nitric oxide synthase activation, leading to vasodilation and angiogenesis, thereby preventing vascular re-occlusion in ischemia-reperfusion injury. Moreover, the mechanical forces generated by cavitation work synergistically with localized oxygen therapy to reduce interstitial H_2_O_2_ levels and decrease the expression of caspase-3, NF-κB, TNF-α, and IL-6, thereby improving the inflammatory response (Ho et al., 2023). This demonstrates that the mechanical forces from cavitation can modulate endothelial cell metabolism, influencing the expression of inflammation-related factors, and potentially affecting the activation or inhibition of the NLRP3 inflammasome complex. Based on this, we hypothesize that the mechanical forces generated by cavitation are also capable of affecting the activation or inhibition of the NLRP3 inflammasome complex. While studies have focused on the pro-inflammatory effects of the NLRP3 inflammasome activation in response to mechanical injury (Sun et al., 2022), ultrasound-mediated mechanical stimuli may modulate NLRP3 activation. The release of ATP or other danger-associated molecular patterns from damaged cells, as a result of the cavitation, could stimulate the NLRP3 inflammasome. However, the targeted delivery of anti-inflammatory drugs, such as MCC950 using ultrasound-microbubble technology could counteract the inflammatory responses and mitigate the detrimental effects associated with the mechanical stimulation. Elucidating whether sonoporation alters the expression of key proteins involved in pro- or anti-inflammatory signaling pathways, such as the NLRP3 inflammasome complex, ASC, caspase-1, and associated cytokines, is essential to further elucidate the mechanisms of action in both the microbubble technology and the MCC950 intervention. Future studies could explore how specific ultrasound parameters are used to finely tune the mechanotransductive response of ultrasound therapy.

In the selection of ultrasound parameters, the specific ultrasound parameters we used (Mechanical index: 0.99, Frequency: 3 MHz, Pulse repetition frequency: 200 Hz, etc., as detailed in Table 1) were rigorously optimized to maximize the therapeutic efficacy of MCC950 while ensuring minimal off-target effects. This combination was empirically determined in preliminary dose-response and safety studies to effectively induce controlled inertial cavitation of the sulfur hexafluoride microbubbles within the renal microvasculature. Inertial cavitation, characterized by the rapid collapse of microbubbles, generates the necessary shock waves and micro-jets that transiently increase the permeability of endothelial and cell membranes. This mechanism is critical for facilitating enhanced MCC950 penetration into the renal parenchymal cells. Higher MI values could potentially increase drug delivery efficiency further, but also carry a greater risk of vascular damage (Yang and Zhou, 2017; Fletcher et al., 2024). We therefore opted for a moderate MI to strike a balance between efficacy and safety. Conversely, lower MI values failed to induce sufficient cavitation for optimal drug delivery compared to high-dose MCC950 alone. The PRF of 200 Hz, along with a pulse time of 2 ms and an interval time of 2 ms, were selected to ensure sufficient microbubble cavitation without inducing excessive thermal accumulation in the kidney tissue (macroscopic observation and histological analysis confirmed the absence of significant local tissue damage). A higher PRF could lead to faster drug release, but also increases the risk of thermal damage (Bai et al., 2024). In addition, the sulfur hexafluoride microbubbles used in this study, which consist of sulfur hexafluoride gas encapsulated within a phospholipid shell, exhibit characteristics of an inert gas with low solubility and high density (Chong et al., 2018). These attributes confer significant *in vivo* stability and extended circulation time to sulfur hexafluoride microbubbles. Furthermore, the comparatively large molecular size of sulfur hexafluoride results in the formation of microbubbles with thinner walls, rendering these microbubbles more susceptible to rupture under ultrasonic stimulation. When subjected to high-frequency ultrasound oscillation, sulfur hexafluoride microbubbles exhibit significant mechanical effects, culminating in rapid bubble rupture, efficient release of encapsulated substances, and augmented cavitation effects. The findings indicated that the therapeutic efficacy of low-dose MCC950 combined with ultrasound microbubbles surpassed that of low-dose MCC950 combined with ultrasound alone in certain aspects, and was comparable to the efficacy of high-dose MCC950 monotherapy. To sum up, this enhanced efficacy is attributed this to the potent mechanical shock waves generated by transient cavitation and the amplification of cavitation effects by microbubbles, which facilitated improved distribution and penetration of MCC950 in renal tissues.

This therapeutic approach, which integrates ultrasound with microbubbles, has been explored in the context of tumor treatment (Huang et al., 2024; Moore-Palhares et al., 2024), blood-brain barrier opening (McDannold et al., 2024), and a range of other pathological conditions (Yang W. et al., 2023; Yue et al., 2024). Empirical evidence indicates that the targeted destruction of gas-filled microbubbles via ultrasound can mitigate ischemia-reperfusion-induced renal fibrosis and improve renal function (Yang J. et al., 2023). Additionally, studies have utilized ultrasound microbubble-mediated Semaglutide therapy in diabetic cardiomyopathy rats, demonstrating effective cardiac protection, significant attenuation of myocardial fibrosis, and reduction in oxidative stress (Liu et al., 2022). Our previous study also showed that ultrasound combined with microbubbles can promote the therapeutic effect of colchicine on gouty arthritis in rats (Zhang et al., 2025). However, research on this approach in the context of CKD remains limited. Thus, this study is a preliminary, basic science investigation performed on an animal model, representing a crucial first step towards clinical applications. In the present study, ultrasound combined with microbubble therapy not only potentiated the inhibitory effect of low-dose MCC950 on the NLRP3 inflammasome but also enhanced the renal protective effects of low-dose MCC950. Improvement in renal function was primarily evidenced by significant reductions in serum creatinine and urea nitrogen levels, which were comparable to the effects observed with high-dose MCC950 alone. Furthermore, HE and Masson staining analyses corroborated these findings, further validating the efficacy of ultrasound combined with microbubble therapy in MCC950-mediated renal protection. This evidence indicates that low-dose MCC950, in conjunction with ultrasound and microbubbles, can produce therapeutic effects comparable to those achieved with high-dose MCC950, thereby effectively mitigating renal damage and preserving renal function.

## 5 Limitations

Despite the substantial findings of this study, several limitations must be acknowledged. First, the experiments were conducted in small animal groups, which limits statistical power and may not capture the heterogeneity of clinical CKD. Variations in baseline characteristics, such as age and baseline renal function, could also introduce confounding factors. Second, our study evaluated treatment effects only at a single time point. This cross-sectional design prevents us from observing the dynamic processes of renal function recovery or the timeliness of fibrosis reversal. Consequently, we could not assess the long-term prognosis, such as rat survival rates or the incidence of end-stage renal disease. Therefore, the translational value of our findings to clinical settings might be limited by the lack of longitudinal observations. Future studies should incorporate multiple time points (e.g., 1, 2, 4 weeks post-treatment and beyond) to delineate the temporal aspects of treatment response and evaluate long-term outcomes. Third, further exploration is required to optimize the parameters of ultrasound (such as frequency, intensity, and duration) and microbubbles (such as concentration and size), as well as the therapeutic protocols, to achieve optimal treatment outcomes. Additionally, treatment strategies for CKD across various etiologies, pathological states, and clinical stages warrant further investigation. Future research will delve into the mechanisms of ultrasound cavitation and microbubbles in different pathological conditions, elucidating their specific effects on cells and tissues to provide a theoretical basis for optimizing treatment strategies. Finally, integrating other drugs or therapeutic modalities to assess the potential of ultrasound combined with microbubbles in multi-targeted combination therapies could improve treatment efficacy and reduce side effects.

## 6 Conclusion

This study demonstrates that the combination of ultrasound and microbubble therapy can potentiate the inhibitory effect of 5 mg/kg MCC950 on the NLRP3 inflammasome, thereby improving renal function and mitigating renal fibrosis in CKD rats. The efficacy of this combined approach exceeds the outcomes observed with MCC950 alone and is comparable to the effects of the 10 mg/kg MCC950 dose. These findings offer novel insights and methodologies for the application of ultrasound combined with microbubble therapy in renal protection, providing both theoretical and experimental evidence for its potential utility in the treatment of other inflammatory diseases.

## Data Availability

The original contributions presented in the study are included in the article/Supplementary Material, further inquiries can be directed to the corresponding author.
